# FACTORS ASSOCIATED WITH ELECTRONIC WEARABLE DEVICE USE AMONG US OLDER ADULTS

**DOI:** 10.1093/geroni/igac059.1473

**Published:** 2022-12-20

**Authors:** Mengchi Li, Qiwei Li, Chakra Budhathoki, Sarah Szanton, Junxin Li

**Affiliations:** Johns Hopkins, University, Baltimore, Maryland, United States; Johns Hopkins School of Nursing, Baltimore, Maryland, United States; Johns Hopkins University, Baltimore, Maryland, United States; Johns Hopkins University School of Nursing, Baltimore, Maryland, United States; Johns Hopkins University, Baltimore, Maryland, United States

## Abstract

Electronic wearable devices(EWD) have been increasingly used in the adult population. Although health benefits of using EWDs may be appealing through health self-monitoring(e.g., physical activity, heart rate, and sleep), safety alerts(e.g., fall risk detection), or stress reduction(breathing technique), there is a lack of understanding in EWD use among older adults. This cross-sectional study used self-reported data of 3370 older adults aged 65 to 104 years from the Health Information National Trends Survey 2019 and 2020. We examined the prevalence of EWD use and used Logistic Regression Models to identify social factors (gender, race/ethnicity, education, and income) associated with the use of EWDs in older Americans. We found that EWD users increased from 13.3% in 2019 to 17.4% in 2020 in older Americans. After adjusting for age, insurance, smoking, marital-status, chronic conditions, region, and all other social factors of interest, we found that older men(Odds Ratio[OR]= 0.58, 95%Confidence Interval[CI]:0.45, 0.73), Black/African American older adults(OR=1.50, 95%CI: 0.41, 0.68), older adults who did not attend college(OR=0.62, 95%CI: 0.46, 0.83), and those with household income lower than $35,000(OR=0.32, 95%CI: 0.24, 0.46) were less likely to use EWDs. Interestingly, before adjustment, EWD use in Black/African American older adults(OR: 1.02, 95%CI:0.74, 1.34) did not differ from that in White/Caucasian older adults. Our findings suggest inequality of EWDs use in older Americans: those with lower education and income had less access to EWDs and fewer potential health benefits from EWDs. This study highlights the needs of equal access to EWDs and addressing social inequalities among older Americans.

